# Secreted Aspartic Protease Cleavage of *Candida albicans* Msb2 Activates Cek1 MAPK Signaling Affecting Biofilm Formation and Oropharyngeal Candidiasis

**DOI:** 10.1371/journal.pone.0046020

**Published:** 2012-11-06

**Authors:** Sumant Puri, Rohitashw Kumar, Sonia Chadha, Swetha Tati, Heather R. Conti, Bernhard Hube, Paul J. Cullen, Mira Edgerton

**Affiliations:** 1 Department of Oral Biology, University at Buffalo, Buffalo, New York, United States of America; 2 Nuclear Agriculture and Biotechnology Division, Bhabha Atomic Research Centre, Mumbai, India; 3 Department of Medicine, Division of Rheumatology and Clinical Immunology, University of Pittsburgh, Pittsburgh, Pennsylvania, United States of America; 4 Department of Microbial Pathogenicity Mechanisms, Leibniz Institute for Natural Product Research and Infection Biology - Hans Knoell Institute Jena, Jena, Germany; 5 Friedrich Schiller University, Jena, Germany; 6 Department of Biological Sciences, University at Buffalo, Buffalo, New York, United States of America; King's College London Dental Institute, United Kingdom

## Abstract

Perception of external stimuli and generation of an appropriate response are crucial for host colonization by pathogens. In pathogenic fungi, mitogen activated protein kinase (MAPK) pathways regulate dimorphism, biofilm/mat formation, and virulence. Signaling mucins, characterized by a heavily glycosylated extracellular domain, a transmembrane domain, and a small cytoplasmic domain, are known to regulate various signaling pathways. In *Candida albicans*, the mucin Msb2 regulates the Cek1 MAPK pathway. We show here that Msb2 is localized to the yeast cell wall and is further enriched on hyphal surfaces. A *msb2*Δ*/*Δ strain formed normal hyphae but had biofilm defects. Cek1 (but not Mkc1) phosphorylation was absent in the *msb2Δ/Δ* mutant. The extracellular domain of Msb2 was shed in cells exposed to elevated temperature and carbon source limitation, concomitant with germination and Cek1 phosphorylation. Msb2 shedding occurred differentially in cells grown planktonically or on solid surfaces in the presence of cell wall and osmotic stressors. We further show that Msb2 shedding and Cek1 phosphorylation were inhibited by addition of Pepstatin A (PA), a selective inhibitor of aspartic proteases (Saps). Analysis of combinations of Sap protease mutants identified a *sap8Δ/Δ* mutant with reduced MAPK signaling along with defects in biofilm formation, thereby suggesting that Sap8 potentially serves as a major regulator of Msb2 processing. We further show that loss of either Msb2 (*msb2Δ/Δ*) or Sap8 (*sap8Δ/Δ*) resulted in higher *C. albicans* surface β-glucan exposure and *msb2Δ/Δ* showed attenuated virulence in a murine model of oral candidiasis. Thus, Sap-mediated proteolytic cleavage of Msb2 is required for activation of the Cek1 MAPK pathway in response to environmental cues including those that induce germination. Inhibition of Msb2 processing at the level of Saps may provide a means of attenuating MAPK signaling and reducing *C. albicans* virulence.

## Introduction

*Candida albicans* is an opportunistic human fungal pathogen responsible for a wide variety of infections in immunocompromised patients as well as oropharyngeal candidiasis (OPC) in medically compromised individuals and denture users. Virulence in *C. albicans* has been traced to the formation of invasive hyphal filaments that bind to and penetrate host cells, to the formation of compact mats/biofilms that show high levels of resistance to antibiotics, and to interactions with the host immune system through cell-surface proteins. The ability of *C. albicans* biofilms to adhere to medical and prosthetic devices contributes to successful colonization of specific sites that include the oral cavity. These virulence determinants are regulated by signal transduction pathways in response to niche-specific environmental cues encountered during colonization of the host (reviewed in [1]). Among the pathways that regulate virulence in *C. albicans* are mitogen-activated protein kinase (MAPK) pathways, which are canonical signaling pathways involved in the regulation of cellular differentiation and proliferation in eukaryotes (reviewed in [2]). Four MAPK pathways have been identified in *C. albicans*: the cell wall integrity (Mkc1) pathway, the high osmolarity glycerol response (HOG) pathway, the cell morphogenesis/hyphal formation (Cek1) pathway, and the mating (Cek2) pathway (reviewed in [3]). Each of these pathways regulate a different aspect of *C. albicans* cellular responsiveness, functioning as a master-regulator of cell fate.

**Figure 1:**
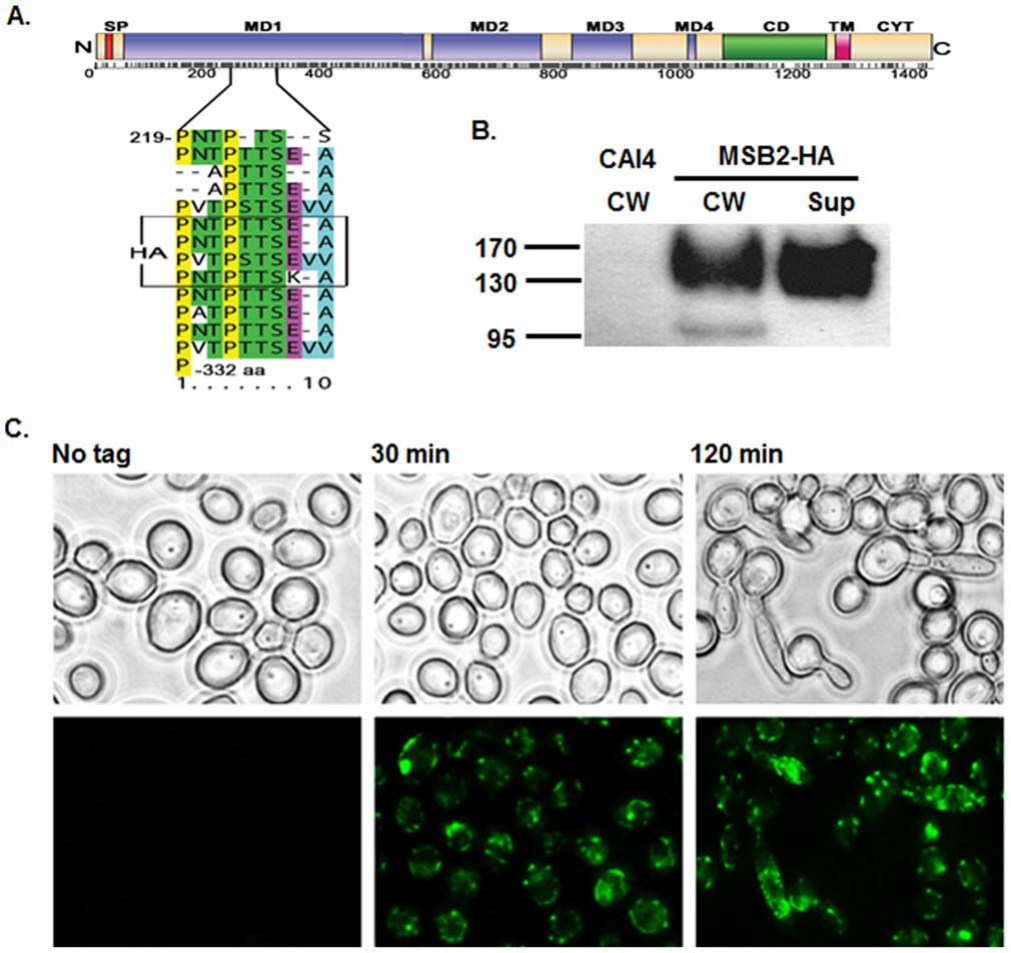
Construction and detection of HA-tagged Msb2. **A.** Schematic depiction of domains of Msb2 protein and the site of introduction of an HA tag. Shown are the N-terminal signal sequence (SP, red), mucin repeat domains (MD, purple), predicted cleavage domain (CD, green), the transmembrane domain (TM, pink), and the cytoplasmic tail (CYT). N and C terminii are denoted by N and C respectively, with numbers below corresponding to amino acid residues. The expanded region details the site of introduction of the HA tag. **B.** Immunoblotting with anti-HA antibody shows the presence of HA-tagged Msb2 in the cell wall (CW) and supernatant (Sup) of the Msb2-HA strain. **C.** Msb2 is surface localized. Cells cultured in YNB media were shifted to 37°C induce germination, and removed at the indicated time intervals and probed with anti-HA Alexa Flour 488 conjugate for visualization of Msb2-HA (lower panel) and directly compared with phase contrast images (upper panel). Msb2 was surface localized and was especially dense on distal surfaces of hyphae.

Initial studies established a role for the Cek1 pathway in starvation-specific hyphal differentiation and growth of serum-induced mycelial colonies [4]. However, Cek1 plays a broader role in establishing fungal infection, as the *cek1Δ/Δ* mutant had attenuated virulence in a murine model of systemic candidiasis [4]. The Cek1 pathway was further implicated in being responsive to yeast quorum sensing and to cell wall damaging agents [5], [6]. Furthermore, the Cek1 pathway responds to glycosylation defects in the cell wall [7] and modulates β-glucan exposure on the cell surface that in turn affects the extent of Dectin-1 mediated immune response against *C. albicans* cells [8]. Yi et al showed a role for the Cek1/Cek2 pathway in biofilm regulation in an a/a mating type of *C. albicans* by mutational analysis [9]. Thus signal transduction through the Cek1 pathway is responsible for the maintenance of a wide variety of virulence traits in *C. albicans*.

Signaling molecules modulating filamentation are highly conserved among fungi [10]. In *Saccharomyces cerevisiae*, the Kss1 MAPK pathway controls filamentous growth and is closely related to the *C. albicans* Cek1 pathway [11], [12]. Msb2 is the head receptor protein that feeds into the signaling cascade of the *S. cerevisiae* Kss1 MAPK pathway, and is structurally very similar to *C. albicans* Msb2. Msb2 has since been identified as the head sensor protein of the Cek1 MAPK pathway and is needed for the successful execution of most Cek1 mediated functions in *C. albicans*, such as invasive growth and cell wall biosynthesis [6]. However, the mechanism by which Msb2 transmits signals to the Cek1 pathway is not known.

Msb2 belongs to a class of glycoproteins called signaling mucins. These mucins are characterized by a highly glycosylated extracellular domain containing a Ser/Thr/Pro-rich mucin homology domain (MHD) [13], one transmembrane region, and a cytoplasmic domain that connects to and regulates cytosolic signaling molecules [14]. Signaling mucins are processed within their extracellular domains, which results in the release of the external glycodomain from the cells [15]. For *S. cerevisiae* Msb2, processing is required for MAPK activation [16]. The aspartic protease Yps1 was shown to proteolytically cleave ScMsb2, and in the absence of Yps1, the Kss1 MAPK pathway activation was inhibited, underscoring the functional importance of this cleavage in the signaling process. Mass spectroscopy analysis of the *C. albicans* secretome identified a soluble form of Msb2 with peptide fragments originating from within the cleavage domain, suggesting post-translational processing of Msb2 [17]. Moreover, a recent study showed that the heavily glycosylated extracellular domain of Msb2 is shed into the medium and that Cek1 phosphorylation is absent in cells harboring truncated versions of Msb2 [18]. But whether *C. albicans* Msb2 regulates Cek1 signaling in a cleavage-dependent fashion is not well understood.

Here we show that the extracellular domain of Msb2 is shed in response to specific environmental cues, and that this shedding is tightly linked to Cek1 phosphorylation. Furthermore, we identify a role for the secreted aspartic protease (Sap) family of proteins in the proteolytic processing and cleavage of Msb2. We also present evidence that processing of Msb2 is required for facilitation of various Cek1 signaling-mediated virulence traits, such as biofilm formation and exposure of cell surface immunogens. The results presented here allow us to postulate that Msb2 is required for *C. albicans* virulence because its proteolytic processing is necessary for the activation of the Cek1 MAPK pathway.

## Results

### *Candida albicans* Msb2 is cell surface localized and a cleavage product of Msb2 is shed from the cells into the media

*Candida albicans* Msb2 is a highly glycosylated protein with a large extracellular region (aa 1–1296) containing four mucin repeat domains (MD), a single transmembrane (TM) domain near its C terminus (aa 1297–1319), and a small cytoplasmic tail (aa 1320–1409) (Figure 1 A). CaMsb2 also has a potential aspartic protease cleavage site (CD) near aa 1200 which suggests that it might have a functional mechanism of signal transduction mediated by its extracellular release. To investigate the possible role of Msb2 in activating the Cek1 cascade in *C. albicans*, an Msb2 deletion mutant and its restoration strain (*msb2Δ/Δ* and *msb2Δ/Δ*+, respectively; Table 1) were constructed. We also made an Msb2-HA strain containing a single internal hemagglutinin (HA) epitope by substitution of four small tandem repeats within the MD1 region of the extracellular glycosylated region of Msb2 (Figure 1 A, box) in order to detect Msb2 cleavage and release. The epitope-tagged version of the protein was fully functional with respect to MAPK activation phenotype (discussed in the next section) and allowed us to test if Msb2′s extracellular domain might be shed from cells. Supernatants were collected from cells cultured under germination conditions (37°C) over 4 hours and probed for the presence of Msb2-HA. Indeed, the Msb2-HA strain released a cleavage product that was detectable by immunoblotting with anti-HA antibody in the cell supernatant and was also found in cell wall fractions (Figure 1 B). The apparent molecular weight range of released Msb2 was similar to the size of cell wall localized protein since the cleaved portion consists of about 1200 amino acid residues (out of 1409 amino acid residues of the full length protein) and contains the majority of the glycosylated regions.

**Table 1:** *C. albicans* strains used in this study.

Strain	Genotype	Reference
**CAI4**	Δ*ura3::imm434*/Δ*ura3::imm434*	[50]
**Msb2-HA**	*Δura3::imm434/Δura3::imm434,Msb2/msb2::FRT/Msb2-HA*	This work
***msb2Δ/Δ***	Δ*ura3::imm434*/Δ*ura3::imm434*, Δ/Δmsb2*::URA*	This work
***cek1Δ/Δ***	*ura3/ura3 cek1Δ::hisG/cek1Δ::hisG*	[4]
***sapΔ/Δ1/2/3***	*sap1Δ::hisG/sap1Δ::hisG sap2Δ::hisG/ sap2Δ::hisG sap3Δ::hisG sap3Δ::hisG*	[43]
***sapΔ/Δ4/5/6***	*sap6Δ::hisG/sap6Δ::hisG sap4Δ::hisG/ sap4Δ::hisG sap5Δ::hisG/sap5Δ::hisG*	[44]
***sap8Δ/Δ***	*Δsap8::hisG/Δsap8::hisG-URA3-hisG*	This work
***sapΔ/Δ9/10***	*sap10Δ::hisG/sap10Δ::hisG sap9Δ::hisG/ sap9Δ::hisG*	[45]
***sap8Δ/Δ*** **+**	*Δsap8::hisG/ Δsap8::hisG-URA3-hisG/SAP8*	This work
***msb2Δ/Δ*** **+**	Δ*ura3::imm434*/Δ*ura3::imm434*, Δ/Δmsb2*::FRT/MSB2*	This work

To confirm the *in vivo* cell surface localization of this protein, yeast and germinated forms of *C. albicans* were visualized by fluorescence microscopy with AlexaFlour 488 conjugated anti-HA antibodies (Figure 1 C). Msb2 was visualized as being distributed predominantly at the surface of all yeast cells examined (Figure 1 C, 30 min). Interestingly, Msb2 was highly enriched at distal hyphal surfaces of germinated cells (Figure 1 C, 120 min) and in some instances hyphal tips appeared to be heavily covered with Msb2. No immunofluorescence was observed in the “no tag” control.

**Figure 2:**
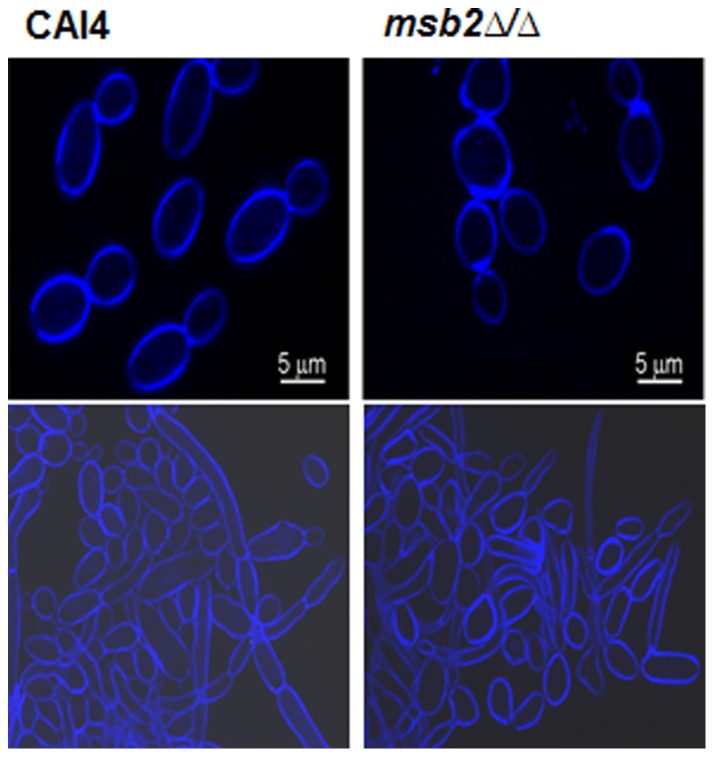
*msb2Δ/Δ* cells show normal hyphae formation. Hyphal formation is unchanged in *msb2Δ/Δ* cells, although cell wall chitin distribution is uneven. W*T* CAI4 and *msb2Δ/Δ* cells were grown overnight grown in YPD, then incubated at 30°C to maintain yeast cells (top) or 37°C for hyphal growth (bottom), and stained with Calcofluor white (CFW) for visualization of cell wall chitin.

Since Cek1 may influence hyphal formation and cell wall composition [4], [7], [8], [19], we examined cell morphology and chitin deposition by fluorescent microscopy in *msb2Δ/Δ* cells compared with CAI4 cells. Aberrant chitin deposition characterized by bunching of chitin at the septa in both yeast cells and hyphae was observed in cells lacking Msb2 (Figure 2, top), suggesting the possibility of septation defects which may account for the slightly delayed growth rate (by about 15%, data not shown) of *msb2Δ/Δ* cells. However, *msb2Δ/Δ* cells readily formed true hyphae, and the extent of germination did not differ between *msb2Δ/Δ* and wild type (WT) CAI4 cells (Figure 2, bottom).

### Msb2 is shed from cells in response to environmental conditions and shedding correlates with Cek1 phosphorylation and germination

**Figure 3:**
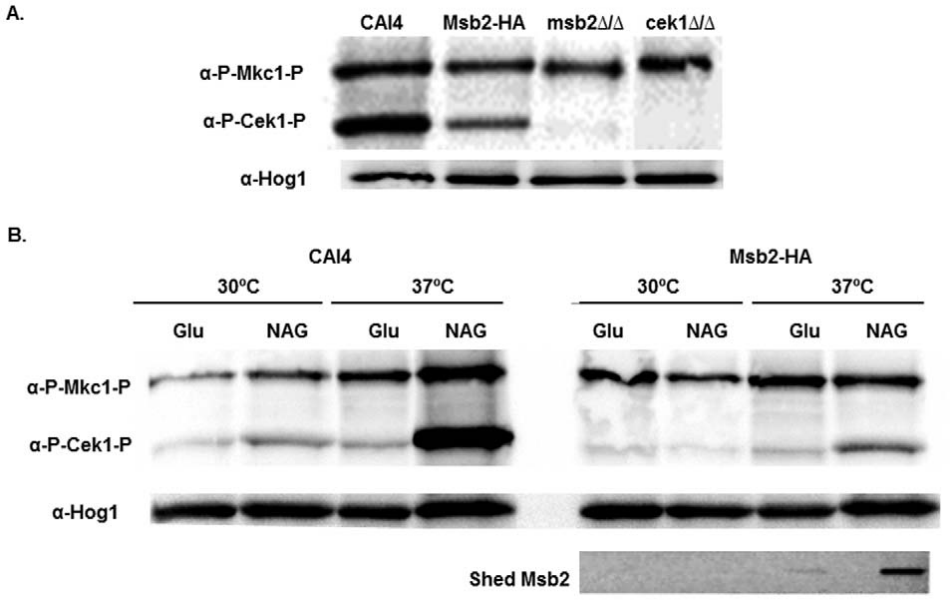
Msb2 is needed for Cek1 phosphorylation and is shed accompanying Cek1 phosphorylation in response to environmental cues. **A.** Cek 1 phosphorylation is not detected in *msb2Δ/Δ* cells. All strains were cultured in prewarmed YNB media with 1.25% *N*-Acetyl-D-Glucosamine (NAG) as carbon source and incubated at 37°C (germination conditions) for 4 h. Total cellular protein (20 µg) from cell lysates were immunoblotted with α-phospho p42/44 MAPK ERK1/2 Thr202/Tyr204 rabbit monoclonal antibody. Mkc 1 phosphorylation is intact while Cek 1 phosphorylation is not evident in *msb2Δ/Δ* cells and in *cek1Δ/Δ* cells, the latter used as a negative control. Immunoblot with α-Hog is shown below as a loading control. **B.** Msb2 shedding and Cek1 phosphorylation is differentially induced by temperature and carbon source conditions. Overnight cultures (CAI4, left; Msb2-HA, right) were diluted and transferred to YNB media prewarmed to 30°C or 37°C, with NAG or Glucose (Glu) as a carbon source and incubated at the respective temperatures for 4 h. To determine Msb2 shedding in Msb2-HA cells, cell supernatants were slot-blotted with anti-HA antibody and detected with ECL plus chemiluminescence. Whole cell lysates (20 µg) were immunoblotted for Cek1 phosphorylation under the same conditions. Cultures subjected to both elevated temperature (37°C) and NAG had highest Msb2 shedding and Cek1 phosphorylation. Immunoblot with α-Hog is shown as a loading control.

Msb2 is known to activate the Cek1 pathway in response to inhibition of glycosylation and cell wall stress [6]. We examined the possibility that Msb2 has a broader role in response to environmental cues. To confirm that *msb2Δ/Δ* cells could not activate the Cek1 pathway, we compared Cek1 phosphorylation in cells lacking Msb2 with WT cells (Figure 3 A). As expected, *msb2Δ/Δ* cells did not show phosphorylation of Cek1, while activation of a related MAPK pathway, the cell wall integrity (Mkc1) pathway [20] remained intact. Also, the Msb2-HA strain containing a single HA tagged Msb2 allele was fully functional in terms of its ability to phosphorylate Cek1 (Figure 3 A). As expected, no Cek1 phosphorylation was observed in *cek1Δ/Δ* cells; and Hog levels were equal among the strains and were used as a loading control (Figure 3 A).

Cleavage of Msb2 is required for MAPK activation in *S. cerevisiae* and the protein is shed post-cleavage into the supernatant [16]. The relatively high density of Msb2 on germ tubes (Figure 1 C, 120 min) suggested that hyphal inducing conditions might be a signal for Msb2 shedding. Thus, we investigated the ability of known hyphae inducers including elevated temperature (37°C) and *N*-Acetyl-D-glucosamine (NAG) as a carbon source [21], [22] to induce Cek1 phosphorylation and Msb2 shedding. In CAI4 cells, combining 37°C temperature shift and NAG as carbon source induced optimal Cek1 phosphorylation; while cells exposed to either 37°C or NAG alone had a weaker phosphorylation response at the 4 h time point examined (Figure 3 B, left). To examine whether Cek1 phosphorylation was accompanied by Msb2 release, we tested the HA-tagged Msb2 strain under the same conditions (Figure 3 B, right). Msb2-HA displayed an identical phenotype to WT cells in that the strongest Cek1 phosphorylation was induced by a combination of 37°C and NAG (Figure 3B, right). Significantly, Msb2 shedding into the media supernatant was strongest in those cells exposed to a combination of 37°C and NAG, while less release occurred under conditions inducing weaker Cek1 phosphorylation (37°C or NAG alone). Cells cultured in non-germination conditions (30°C + Glu) had neither Msb2 shedding nor Cek1 phosphorylation, showing that both Msb2 release and Cek1 phosphorylation are specifically induced by the combination of a shift to higher temperature and NAG as a carbon source.

**Figure 4:**
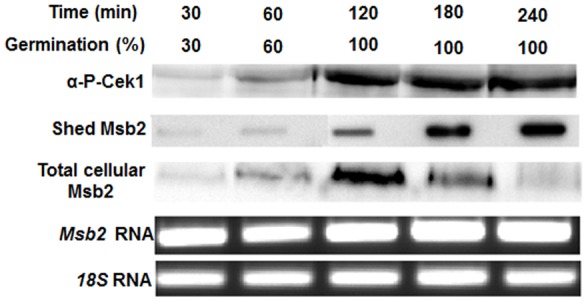
Cek1 phosphorylation and percentage of germination parallel Msb2 shedding. Overnight cultures of Msb2-HA cells were grown under germination conditions over 4 h and analyzed at indicated time points. Germination percentage was calculated among 200 cells observed at 10X magnification. Whole cell lysates (20 µg of protein) were immunoblotted for detection of total cellular Msb2 protein and Cek1 phosphorylation and cell supernatant was slot-blotted for detection of Msb2 shedding. Total RNA (1 µg) isolated from cells was reverse transcribed and used for amplification using *MSB2* specific primers along with *18S* RNA specific primers as control. Cek1 phosphorylation closely paralleled percent germination and Msb2 shedding, which was not a result of increased transcription of *MSB2* over the first 120 min.

We next examined the temporal relationship of *C. albicans* germination with Msb2 shedding and Cek1 phosphorylation under our defined temperature and carbon source conditions. Cek1 phosphorylation closely paralleled Msb2 shedding with both events reaching a peak at about 3 hours (Figure 4). Germ-tube formation also paralleled with these events and peaked at 3 hours under these conditions (Figure 4). We also examined total cellular levels of Msb2 protein and *MSB2* transcript under the same conditions. Total cell associated Msb2 protein levels increased up to 2 hours and then were reduced concomitantly with an increase in Msb2 release into the media (Figure 4). Transcriptional levels of *MSB2* RNA (analyzed by densitometry; data not shown) remained relatively constant up to 120 min and then increased by 1 fold (Figure 4); most likely as a result of higher demand for Msb2 protein localized on the increased surface area of growing germ tubes as shown in Figure 1 C. Taken together, these results suggest that cell-associated Msb2 protein levels do not regulate Cek1 phosphorylation or germination; instead these processes depend upon the extent of Msb2 shedding. Thus, Msb2 processing and shedding is not constitutive, but is closely linked with germination and activation of the Cek1 pathway.

### Msb2 is shed in response to a range of environmental cues during planktonic and solid surface growth

**Figure 5:**
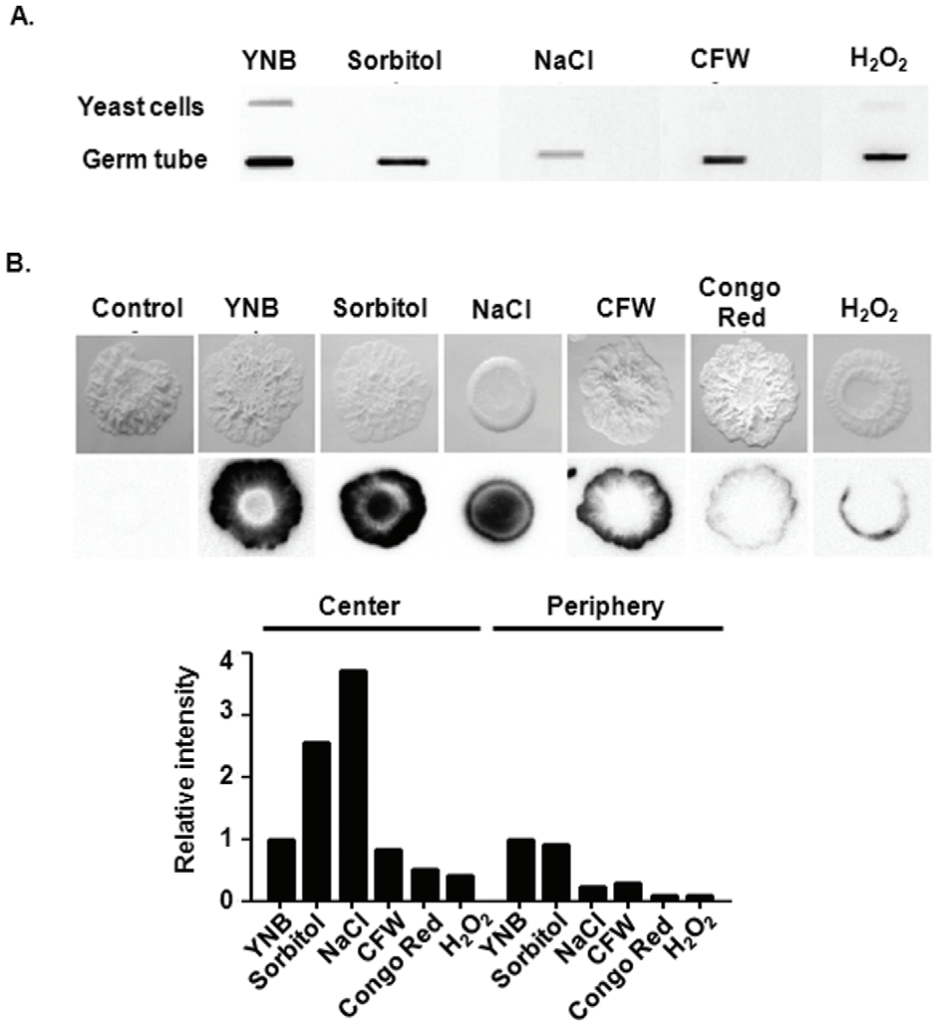
Msb2 shedding responds to a wide variety of environmental cues both under liquid culture and solid surface growth conditions. **A.** Overnight cultures of Msb2-HA cells were grown under germination or non-germination conditions, washed, and resuspended in fresh media under different stress conditions: osmotic stress (1 M Sorbitol, 1 M NaCl), cell wall stress (100 µg/ ml CFW), and oxidative stress (5 mM H_2_O_2_) for 1 h. Msb2 shedding was determined by immunoblotting of cell supernatants. **B.** Msb2 shedding on solid surfaces differs between colony centers and peripheries. Msb2-HA strain was grown on nitrocellulose membrane overlaid on YNB + 1.25% NAG +1% agar containing the stress inducing agents in (A), as well as 100 µg/ ml of Congo red. After 3 days, colony morphology was documented and nitrocellulose membranes were washed, blocked, and immunoblotted with anti-HA antibody for Msb2 shedding determination (top). The intensity of shed Msb2 was determined using Image J software (bottom). The relative Intensity was calculated by dividing intensity of shed Msb2 under different stress conditions with shed Msb2 under YNB. Control (far left) depicts colony morphology in WT cells that do not carry a tagged version of Msb2.

To determine whether Msb2 shedding is responsive to other environmental cues in addition to those that induce germination, we investigated shedding of Msb2 in response to various stress conditions. As shown in Figure 5 A, addition of sorbitol and NaCl (osmotic stressors), Calcoflour white (cell wall stressor), and peroxide (oxidative stressor) led to a reduction of varying degrees in Msb2 shedding for both yeast cells and germ tubes that were grown planktonically. However, the extent of reduction was difficult to visualize for yeast cells, primarily because of the low levels of shedding seen in yeast cells as compared to the germ tubes.

As planktonic cells often differ from cells that are a part of a microbial community, we investigated Msb2 shedding in response to various stresses with cells grown as colonies on a solid surface (Figure 5 B). *C. albicans* colonies are bipartite, where the central region is more characteristic of the yeast form with some pseudohyphae and hyphae, while the peripheral region is mainly composed of pseudohyphae and hyphae [23]. Interestingly, colony peripheries showed a shedding pattern distinctive from the colony center. All stressors reduced peripheral shedding, with the least reduction in the presence of sorbitol and maximal reduction in the presence of Congo red (another cell wall stressor besides calcoflour white) and peroxide. In contrast, osmotic stressors led to an increase in central shedding. Thus, Msb2 shedding responds uniquely to various stress conditions, and further depends upon whether cells are grown planktonically or in contact with a solid surface.

### Proteolytic processing of Msb2 is required for its shedding and activation of Cek1 pathway

**Figure 6:**
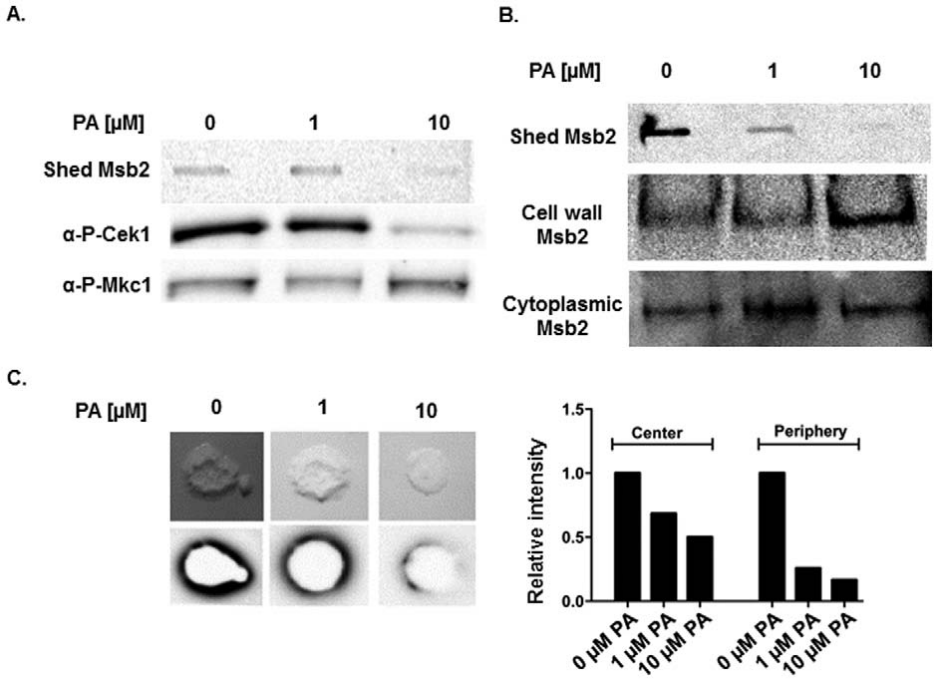
Msb2 shedding and Cek1 phosphorylation are inhibited in the presence of Pepstatin A (PA). Overnight cultures of Msb2-HA cells were grown under germination conditions for 1 h (**A**) or 4 h (**B**) in the presence of indicated concentrations of Pepstatin A (PA) and were analyzed by immunoblotting. **A.** Msb2 shedding in cell supernatants and Cek1 phosphorylation in total cell lysates were inhibited in a dose dependent manner by PA. **B** Msb2 levels in cell wall and cytoplasmic fractions remained unchanged or increased in cell walls upon treatment with PA, showing that Msb2 remains localized to cell surface but is not shed in the presence of PA. **C.** Msb2-HA strain was grown on nitrocellulose membrane overlaid on YNB +1.25% NAG +1% agar for 3 days in the presence of indicated amounts of PA and Msb2 shedding was analyzed as for Figure 5 B. Msb2 shedding from colonies on solid surfaces was inhibited by PA in a dose dependent manner.

We next asked whether post-translational proteolytic processing of Msb2 is required for Msb2 shedding and activation of Cek1 phosphorylation. The *S. cerevisiae* Msb2 ortholog is processed by Yps1, a glycosylphosphatidylinositol (GPI) -aspartic protease of the yapsin family of proteases [24]. Release of its so-called inhibitory extracellular domain is necessary for MAPK activation [16]. However, the protease(s) responsible for Msb2 processing in *C. albicans* are not known. The *C. albicans* genome contains 10 secreted aspartic protease genes, *SAP1* through *SAP10* [25]–[27]. To begin to determine whether members of this protease family are required for Msb2 processing, we analyzed Cek1 activation in cells exposed to Pepstatin A (PA), a specific inhibitor of aspartic proteases, for 1 hour. As shown in Figure 6 A, addition of 10 µM PA to cells abolished both Msb2 shedding and Cek1 phosphorylation. This effect was specific for the Cek1 pathway as the phosphorylation of Mkc1 remained unaffected. At a 4 hour time point (Figure 6 B), addition of PA abolished Msb2 shedding in a dose-dependent manner and resulted in accumulation of Msb2 in cell-wall fractions. Internal Msb2 levels, both cell wall and cytoplasmic, did not decrease under the given conditions. Hence, PA had no effect on total Msb2 protein levels but specifically inhibited Msb2 release. Cells grown on a solid surface in media containing PA also showed substantial dose dependent reduction in Msb2 secretion, both at the center and periphery of the colonies (Figure 6 C). This shows that proteolytic processing of Msb2 occurs not only under planktonic growth, but also during growth on solid surface.

**Figure 7:**
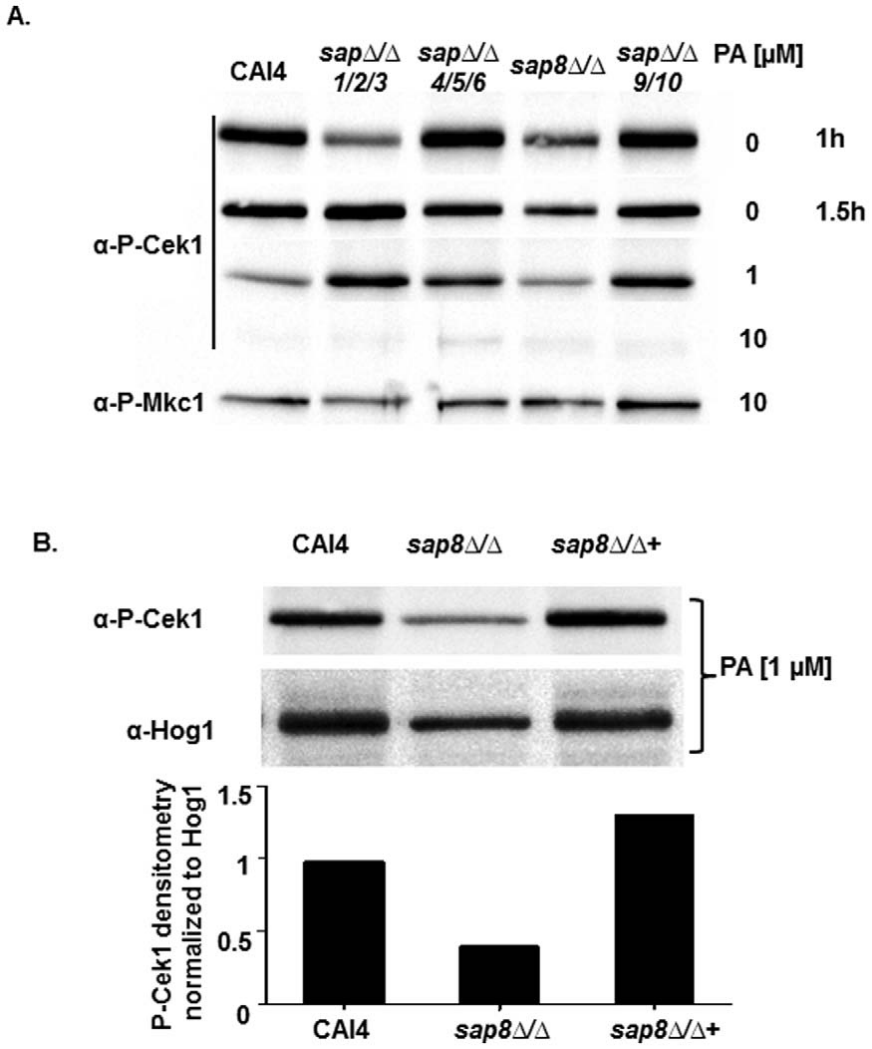
Potential role of Saps in Cek1 phosphorylation. **A.** Cek1 phosphorylation was determined by immunoblotting with CAI4 and with various *SAP* deletion strains, grown under germination conditions with (1 h) or without (1 h and 1.5 h) the indicated concentrations of PA. The *sap8Δ/Δ* strain had reduced Cek1 phosphorylation, which was further decreased in the presence of 1 µM PA. All strains had complete inhibition of Cek1 phosphorylation in the presence of 10 µM PA. **B.** Cek1 phosphorylation was determined by immunoblotting using CAI4, *sap8Δ/Δ*, and *sap8Δ/Δ*+ cells grown under germination conditions in the presence of 1 µM PA for 1 h. Cek1 phosphorylation levels were quantitated by densitometry and normalized to Hog1. The *sap8Δ/Δ*+ strain had higher levels of Cek1 phosphorylation as compared to *sap8Δ/Δ*, indicating the effect to be gene specific.

The efficacy of PA in inhibiting Msb2 shedding, taken together with its high specificity of inhibition towards aspartic proteases, suggested that Sap proteins might be responsible for Msb2 processing and release from cells. Moreover, the cleavage domain of *S. cerevisiae* Msb2 and *C. albicans* Msb2 are conserved, indicating that they might be processed by similar (evolutionarily conserved) proteases. Homology predictions showed that *C. albicans* Sap9 and Sap10 are most closely related to ScYps1 [26] and therefore the most likely candidates to be involved in CaMsb2 processing. However, recent regrouping of *C. albicans* Sap proteins into clades, based on their physiological properties and substrate specificities [28], raised the possibility that Saps1-3, Sap8, and Saps9-10 might all be equally probable candidates for their potential role in Msb2 processing. Therefore, we examined combinations of *C. albicans* Sap mutants for their ability to phosphorylate Cek1 in response to 37°C and NAG. We found that Cek1 phosphorylation after 1 h was reduced in the *sap1Δ sap2Δ sap3Δ* triple mutant as well as in the *sap8Δ/Δ* mutant, but not in *sap4Δ sap5Δ sap6Δ* triple or *sap9Δ sap10Δ* double mutants (Figure 7 A). Cek1 phosphorylation in the *sap1Δ sap2Δ sap3Δ* triple mutant was however more similar to the other *sap* mutants after 1.5 hours, whereas the *sap8Δ/Δ* strain still had reduced Cek1 phosphorylation levels at this time point. Addition of 1 µM PA to the media resulted in a decrease in Cek1 phosphorylation response in *sap8Δ/Δ* cells as well as CAI4 after 1 h of treatment, while other *sap* mutants were unchanged. Strikingly, increasing the concentration of PA to 10 µM resulted in a complete loss of Cek1 phosphorylation in the WT as well as in all *sap* strains (Figure 7 A) while Mkc1 phosphorylation remained unaffected, thus underscoring the specificity of the Cek1 pathway in terms of its dependence on Msb2 processing/cleavage. Among our *sap* mutant strains, *sap8Δ/Δ* appeared to be most affected in regards to loss of Cek1 phosphorylation in response to germination conditions in the presence of low doses of PA. We examined whether this was gene specific using the *SAP8* complemented strain (*sap8Δ/Δ*+) and found that reduction in the levels of phosphorylation in *sap8Δ/Δ* was restored in the *sap8Δ/Δ*+ strain (Figure 7 B, top), suggesting that loss of *SAP8* makes cells less responsive to Cek1 phosphorylation via Msb2 processing. In fact, densitometry analyses (Figure 7 B, bottom) of these strains normalized to Hog1 showed higher levels of Cek1 phosphorylation for the *sap8Δ/Δ*+ strain compared to WT in the presence of 1 µM PA, which may be a result of over-expression of *SAP8* in the complementation strain and/or relative reduction of phosphorylation of the WT in the presence of 1µM PA (as shown in Figure 7A). However, the considerable functional redundancy among the Sap family members does not rule out a contribution for Msb2 processing by other Saps, especially since compensatory up-regulation of remaining *SAP*s may occur in triple *SAP* gene deletion mutants. Therefore, we took a functional approach to examining the role of Saps by comparison of the phenotypes of *SAP* deletion mutants with that of *msb2Δ/Δ*. We expected that strains lacking the most important Sap proteins involved in Msb2 processing would most closely phenocopy deletion of Msb2.

### Msb2 processing is involved in biofilm formation

**Figure 8:**
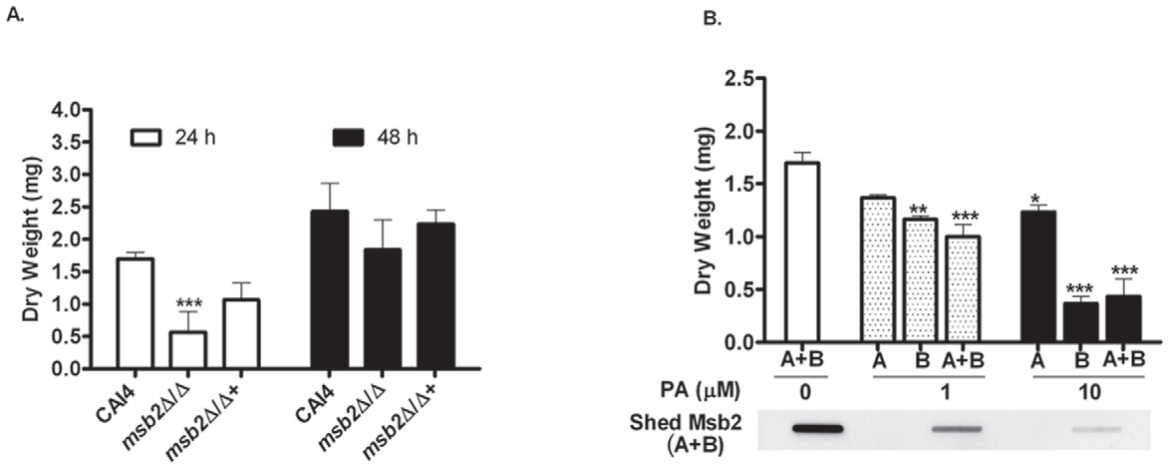
Msb2 deletion reduces biofilm formation. Strains were grown in 24 well plates in YNB +2% glucose at 37°C. Biofilms were harvested at 24 h and/or 48 h and quantitated by dry weight. Measurements of biofilm mass were done in triplicate wells and from at least two independent experiments. **A.** The *msb2Δ/Δ* strain had reduced (p<0.05) biofilm formation at 24 h compared to CAI4 and *MSB2* restoration (*msb2Δ/Δ*+) strains. **B.** Msb2 shedding and biofilm formation are reduced in the presence of PA. Msb2-HA cells were incubated with indicated concentrations of PA for 2 h (biofilm adhesion phase, denoted as A), 24 h (biofilm expansion phase, denoted as B), and 2+24 h (denoted as A+B), during biofilm formation. Biofilm mass was calculated (top) as described for **A** and biofilm supernatants (A+B conditions) were collected and analyzed for Msb2 shedding by slotblotting (bottom).

Based on the dependence of the Cek1 pathway on Msb2 for its activation, we hypothesized that Msb2 may have a role in biofilm formation in *C. albicans*. We investigated the extent of biofilm formation in the *msb2Δ/Δ* strain. As seen in Figure 8 A, there was a 3-fold decrease in biofilm mass after 24 hour proliferation in the absence of Msb2 as compared to the WT. However by 48 hour, *msb2Δ/Δ* biofilm formation increased to WT levels. The restoration strain with a copy of *MSB2* successfully formed biofilm mass similar to WT levels at both 24 and 48 hours.

Karunanithi *et al*. established a potential relationship between shedding of *S. cerevisiae* mucin protein Flo11/Muc1 and biofilm formation [29]. To investigate if a similar relationship exists between Msb2 shedding and biofilm formation in *C. albicans*, we investigated the extent of biofilm formation, both in the presence of PA, as well as with various *SAP* mutants. Biofilm formation by Msb2-HA cells was impaired in the presence of PA when added both in the initial adherence phase (A at 2 hour) and the expansion phase (B at 24 hour) as compared to the control (no PA) (Figure 8 B). Inhibition of biofilm formation was most significant at 10 µM PA, the concentration that also led to complete loss of Msb2 shedding (Figure 6 A, B). To establish a more direct relationship between shedding and biofilm formation, we examined shedding from the biofilm by densitometry analyses of blotted biofilm supernatants. Inhibition of Msb2 shedding in biofilms grown on surfaces containing 1 and 10 µM PA (Figure 8 B, bottom) was linearly proportional with the decrease in the biofilm mass (Figure 8 B, top). Based on these results, a linear correlation between Msb2 shedding and biofilm formation capacity was observed, showing a link between Msb2 shedding during biofilm formation and the extent of the biofilm formed.

**Figure 9:**
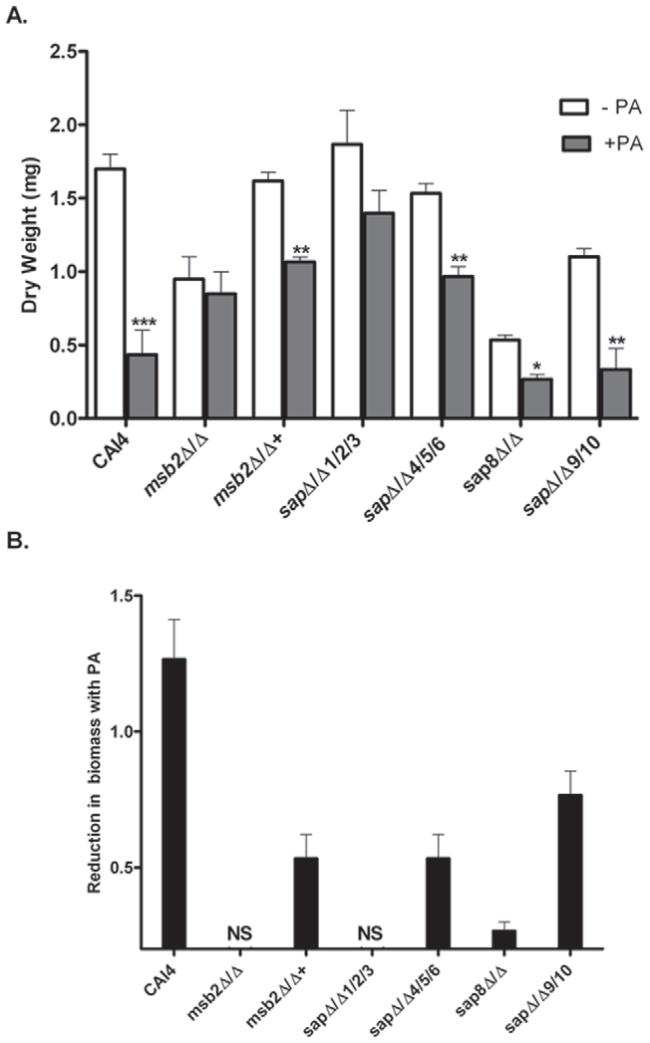
Msb2 and its proteolytic processing are involved in biofilm formation. Biofilm mass was compared in the absence (white bars) or presence (black bars) of 10 μM PA in CAI4, *msb2Δ/Δ*, *msb2Δ/Δ*+, and various *SAP* deletion cells (A), and the absolute value of reduction in biofilm formation in the presence of PA was calculated (B). Statistical analysis of differences in biomass for each strain with or without PA at P≤0.05(*), P≤0.01(**) and P≤0.001(***) are shown. CAI4 cells had significantly reduced (P<0.001) biofilm formation in the presence 10 μM PA; however *msb2*Δ/Δ cells produced lower biomass that was not further reduced by PA. Restoration of Msb2 (*msb2*Δ/Δ+) reversed the biofilm formation defect and restored PA sensitivity. Among mutants with equivalent levels of biofilm formation to WT cells, *sapΔ/Δ1/2/3* but not *sapΔ/Δ4/5/6* had further reduction of biomass in the presence of PA. Among *sap* mutants with biofilm formation defects without PA, *sap8Δ/Δ* cells had largest deficit in biomass production that was reduced by PA; while *sap9Δsap10Δ* cells had slightly reduced biofilm formation that was substantially reduced further by PA (B).

Next we tested the ability of PA (10 µM) to inhibit biofilm formation at 24 h among the Sap mutants when compared with WT cells and *msb2Δ/Δ* (Figure 9). We expected that Sap mutants that formed biomass levels comparable to WT cells and remained PA sensitive would not have a role in Msb2 processing; while strains lacking a Sap(s) important for processing would have little further reduction of biofilm in the presence of PA. We found that the presence of 10 µM PA reduced the biofilm mass by 75% in WT cells (Figure 9 A & B). In contrast, *msb2Δ/Δ* cells formed only half the biomass of WT cells, and the presence of PA did not further reduce biomass of *msb2Δ/Δ* cells. The *msb2Δ/Δ+* strain formed WT levels of biofilm mass without PA (Figure 9 A), and biomass was reduced by 50% in the presence of PA (Figure 9 A &B), thus pointing towards a role for Msb2 processing in biofilm formation. Among the Sap mutant strains tested, the *sap1Δ sap2Δ sap3Δ* triple mutant was able to form WT levels of biomass, but this biomass was not significantly reduced by 10 µM PA, thus suggesting a possible role for Sap1, Sap2, and Sap3 mediated processing of Msb2 in biofilm formation. The *sap8Δ/Δ* strain was most similar to the *msb2Δ/Δ* strain in terms of its impaired ability of biofilm formation and well as reduced sensitivity to PA. Both the *sap4Δ sap5Δ sap6Δ* triple mutant and the *sap9Δ sap10Δ* double mutant had significant reduction in biofilm mass in the presence of PA (40 and 70% respectively), although the *sap9Δ sap10Δ* double mutant had impaired biomass production (30%) without PA (Figure 9 A & B), most likely a result of its cell wall defects [26]. Thus, among the Sap mutants examined, the *sap8Δ/Δ* strain was most similar in phenotype to *msb2Δ/Δ*, although our biofilm assays also point to a potential role of Sap1, Sap2, and/or Sap3 in biomass production.

**Figure 10:**
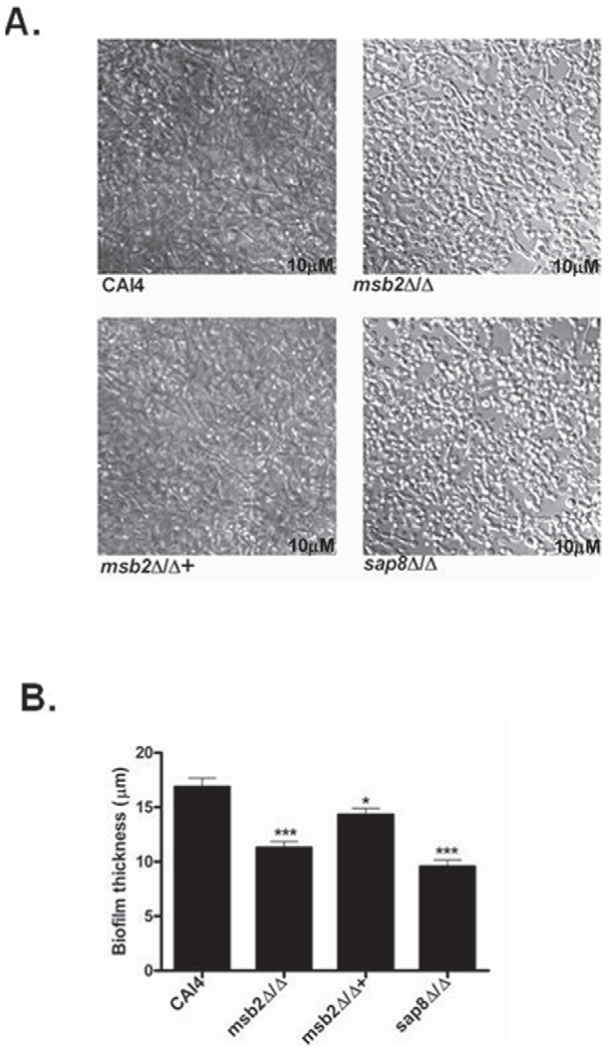
Biofilm architecture and thickness are altered in *msb2*Δ/Δ and *sap8*Δ/Δ strains. **A**. Confocal microscopy images of biofilm formed by *msb2*Δ/Δ and *sap8*Δ/Δ show differences at the morphological level (reduced yeast and hyphal density) as compared to the WT CAI4 and *msb2*Δ/Δ+ strains. Biofilm thickness (**B)** formed by *msb2*Δ/Δ and *sap8*Δ/Δ were significantly (p<0.001) lower than WT, while thickness was nearly restored in the *msb2*Δ/Δ+ strain. Biofilm thickness was determined by merging all *z*-stack images into a three-dimensional projection. Side-view images were obtained using Zieiss LSM image examiner, and thickness was measured for three different areas of the biofilm formed

We also examined the morphology of biofilms formed by WT, *msb2Δ/Δ, msb2Δ/Δ*+, and *sap8Δ/Δ* strains using confocal microscopy (Figure 10). WT CAI4 cells exhibited typical biofilm formation with dense entangled mats of hyphae and yeast cells, as well as layering and stacking of cells (Figure 10 A). However, both *msb2Δ/Δ* and *sap8Δ/Δ* strains formed less dense biofilms (Figure 10 A) and had a significant reduction in biofilm thickness (Figure 10 B); although both strains had abundant hyphae formation and hyphal lengths were similar to those of WT cells. The *msb2Δ/Δ*+ restoration strain formed biofilms that appeared to be phenotypically similar to WT biofilms with nearly WT-level thickness. Defects in the morphology and reduced thickness of biofilm in *msb2Δ/Δ* and *sap8Δ/Δ* strains closely followed the reduction in biofilm mass for these strains, as shown in Figure 9A. Taken together, these results show that proteolytic processing of Msb2 has a role in biofilm formation and maturation, a Cek1 pathway mediated process.

### Msb2 regulates surface β-glucan levels and the ability to establish oral infection

**Figure 11:**
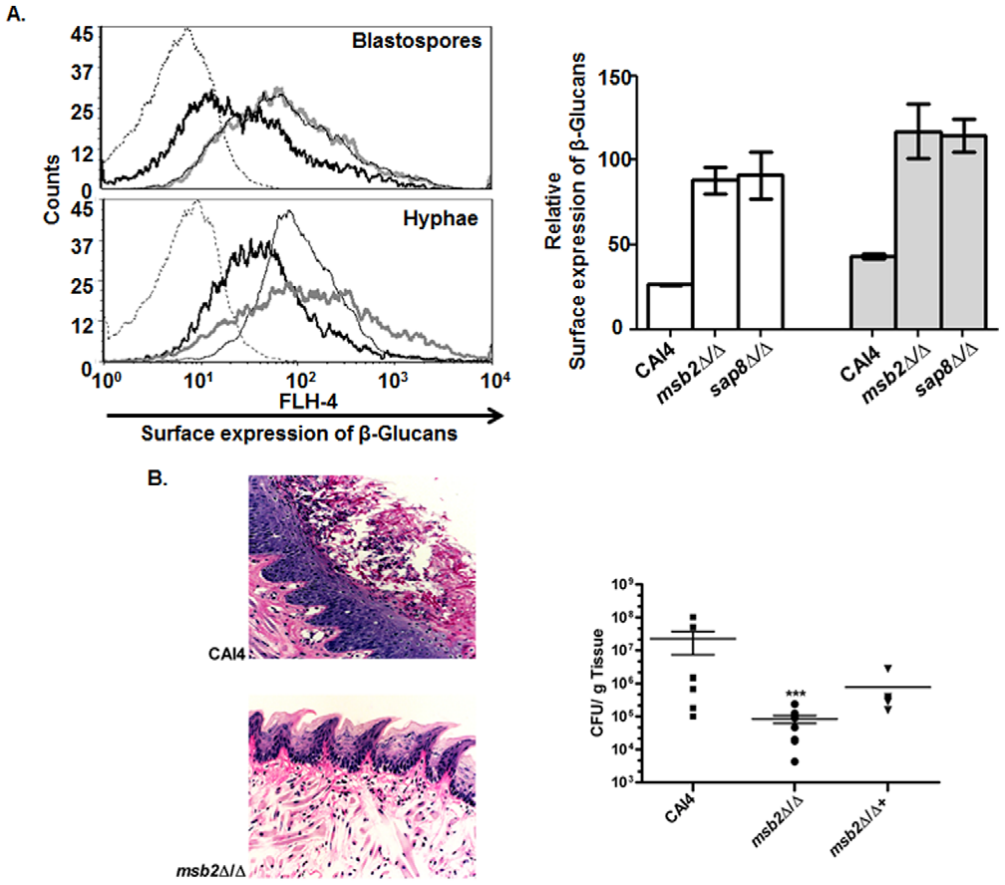
Msb2 is involved in cell surface glucan expression and oropharyngeal candidiasis. A. Surface expression of β-glucan is increased in *msb2Δ/Δ* and *sap8Δ/Δ* cells. CAI4, *msb2Δ/Δ*, and *sap8Δ/Δ* cells were grown under non-germination and germination conditions for 1 h, then cells (3X10^6^) were incubated with anti-β-(1,3)-glucan MAb and Alexa-Fluor 647 secondary labeled Ab. Surface β-glucan levels were analyzed by fluorescence-activated cell sorting (FACS) using FACSCalibur flow cytometer and shown on left: CAI4 (thick black line), *msb2Δ/Δ* (grey line) and *sap8Δ/Δ* (thin black line), and unlabeled WT (dotted line). Quantification (right) of β-glucan surface levels was obtained using FACS for yeast cells (white bars) and germ tubes (grey bars) and showed that *msb2Δ/Δ* and *sap8Δ/Δ* cells had significantly (p<0.05) higher levels of β-glucan. **B.** Fungal colonization (left) and fungal tongue tissue burden (right) were reduced following oral infection by *msb2Δ/Δ* compared to WT. Periodic acid-Schiff stained section of the tongues of infected mice 5 days post infection (left), and CFU/gm tongue tissue (right) were significantly (p<0.01) reduced after infection of *msb2Δ/Δ* cells compared to WT and restoration (*msb2Δ/Δ+*) strains.

The Cek1 pathway controls important virulence traits in *C. albicans*, such as limiting cell surface exposure of β-glucan that allows for immune detection of fungal cells by the host [8] **.** We considered the possibility that this Cek1 mediated effect may also be dependent upon Msb2 mediated phosphorylation, and therefore examined surface β-glucan levels in *msb2Δ/Δ* and *sap8Δ/Δ* strains, the two backgrounds we found to be defective in Cek1 phosphorylation. Both *msb2Δ/Δ* and *sap8Δ/Δ* strains had significantly higher surface β-glucan levels in yeast cells as well as in hyphae compared to the WT strain (Figure 11). Both *msb2Δ/Δ*+ and the *sap4Δ sap5Δ sap6Δ* triple mutant had levels of surface β-glucan that were very similar to the WT strain (data not shown). Thus, Msb2 may have a potential role in establishing virulence in *C. albicans* that can be traced to its proteolytic processing-mediated control of the Cek1 pathway that in turn regulates important cell surface properties including β-glucan exposure.

To further establish a direct role for *msb2Δ/Δ* in virulence, we analyzed its ability to establish oral infection in a murine model of oral candidiasis. Epithelial cell colonization of mice tongues was significantly reduced (p<0.01) from 10^7^ CFU/gm tissue upon infection with WT cells to 10^5^ CFU/gm tissue with *msb2Δ/Δ* cells; however, colonization was restored to WT levels upon infection with the *msb2Δ/Δ+* strain (Figure 11 B, right). Histological evaluation demonstrated that mice infected with WT cells had thick plaques of yeast and hyphae covering the dorsal epithelium that in some regions could be found invading the superficial epithelial layers (Figure 11 B, left). Many regions of the tongues showed a loss of normal epithelial architecture including destruction of the lamina propria as well as extensive neutrophil infiltration. In contrast, mice infected with *msb2Δ/Δ* mutant had virtually no yeast tongue plaques and exhibited normal epithelium with little inflammatory infiltration. Mice infected with WT cells lost weight throughout the time course and demonstrated abnormally low activity levels; however, no significant weight loss or reduction in activity was observed in mice infected with *msb2Δ/Δ* (data not shown). Thus, Msb2 plays a role in the ability of *C. albicans* to establish an oral infection, perhaps through its role in the maintenance of cell wall and surface β-glucans, via its regulation of the Cek1 MAPK pathway activation.

## Discussion

Cleavage of signaling glycoproteins leading to activation of their respective MAPK pathways has begun to emerge as a central theme in MAPK signaling. Certain key features of these proteins, such as the presence of a transmembrane domain (leading to cell surface localization), a heavily glycosylated extracellular domain (responsible for perceiving extracellular signals), and a small cytoplasmic domain (involved in transmission of the perceived signal to the cell interior), are necessary for the functionality of this unique activation mechanism. We provide evidence here for the localization of CaMsb2 on cell surface, with increased density on hyphae (Figure 1 C). Satisfyingly, a recent report has similarly shown that Msb2 from *C. albicans* is shed, and that truncated versions of Msb2 varied in their ability to phosphorylate Cek1, implicating a role for Msb2 processing/cleavage in MAPK activation in *C. albicans* [18]. Our results confirm and extend these important findings and identify proteases that might be involved in signaling mucin regulation in this human pathogen.

Here we show that activation of the Cek1 pathway closely followed the extent of Msb2 shedding into the cell supernatant, with 37°C and NAG as carbon source being the optimal conditions for shedding of Msb2 and phosphorylation of Cek1 (Figure 3 B right). These two processes also closely paralleled the extent of germination (Figure 4). We propose that Msb2 plays an important role in sensing optimal environmental cues for germination in the human host by *C. albicans*. Although the importance of Msb2 in sensing cell wall damage has been established, we observed a reduction in both Msb2 shedding (Figure 5 A) and Cek1 phosphorylation (data not shown) in the presence of cell wall damaging agents like Congo red or CFW, albeit minimal, under liquid culture growth. This is in contrast to the enhanced phosphorylation of Cek1 observed by Roman *et al* [6] in the presence of these agents; however, other growth conditions including the carbon source were different between the two studies. We propose that Msb2 has a variable response to select agents depending on growth conditions, as seen within a microbial community during solid surface growth (Figure 5 B). A recent study suggesting that different domains of Msb2 convey different messages to Cek1 activation [18] could further explain these discrepancies, especially if Msb2 processing rates potentially vary among different set of growth conditions. Regardless, this does not seem to challenge the role of Msb2 as a wall-damage sensor because Msb2 still responds to environmental stress, whether by increase or decrease in the extent of its cleavage and levels of Cek1 phosphorylation. In fact, sensor abilities of Msb2 only have begun to be explored. In this regard, our preliminary results have identified a role for Msb2 in sensing environmental levels of iron that activate Cek1 phosphorylation (data not shown), yet another potential signal for this pathogenic fungi to establish colonization.

We identified PA-mediated inhibition of Msb2 shedding (Figure 6) as strong evidence for the role of *C. albicans* Sap proteins in Msb2 processing since PA has high specificity for aspartic proteases. Although a recent study found PA did not effect Msb2 shedding [18], it is possible that differences in the HA tag placement within the cytoplasmic region of Msb2 may have altered the protein function allowing for its constitutive release; or that differences in growth conditions masked signal-specific Msb2 release. Constitutive secretion of Msb2 is inconsistent with its known role as a sensor protein involved in signaling in response to environmental cues in other closely related organisms. In agreement with our data suggesting a role of environmental conditions in Msb2 shedding, secretome studies in *C. albicans* by the Klis laboratory have shown that Msb2 secretion levels increased in response to temperature [17] and decreased upon cell wall stress mediated by fluconazole [30]. Moreover, regulated secretion of Msb2 by *Fusarium oxysporum* and *S. cerevisiae* was detected in response to solid surface growth [31] and under nutrient starvation conditions [16], respectively. Thus, these and our own studies establish a paradigm for inducible release of this signaling mucin in the fungal world.

Among the ten *C. albicans* Sap family members, we identified Sap8 as the protease most efficient in Msb2 processing under our specific growth conditions. However, *C. albicans* Sap proteins share a high level of homology and it is likely that other Sap proteins have complementary roles in Msb2 processing under other growth conditions. As each *C. albicans* Sap has its unique pH optima and is expressed differentially dependent upon stages and sites of infection [26], [32], [33], we expect that multiple Saps have overlapping roles in Msb2 processing, depending on the *in vitro* growth condition or *in vivo* site of infection. In addition, Saps have different locations within the cell [26], which may allow for differential susceptibilities to Kex2, a protease responsible for activation of Saps [34], pointing to an additional potential means of regulation for Msb2 cleavage. In fact it has been shown that Sap 9 and Sap 10 are not completely inhibited by PA [26] and a very recent report identifies Sap7 as a PA-insensitive aspartic protease [35]. These observations may explain the lack of an absolute effect of PA on Cek1 activation in our hands. Thus, while we clearly show that Sap8 is required for Cek1 phosphorylation (Figure 7 A), we observed variability among our experiments suggesting complementary processing involving other Saps in addition to Sap8. Also, our biofilm studies point to a contributory role for Sap1, Sap2 and Sap3 in Msb2 processing for biofilm formation. It is also possible that expression levels of remaining Saps are altered in strains carrying multiple deletions of individual Saps. Also, we cannot rule out the role of some as yet undiscovered protease in Msb2 processing.

Shed domains of signaling mucins have been shown to have additional functions. For example, the soluble MUC1 has been implicated in suppression of anti-tumor activity by mediating inhibition of the lytic activity of tumor immune cytotoxic T cells or repression of immune cell recruitment at the tumor site (discussed in [36]). We discovered a direct relationship between the extent of Msb2 shedding and the robustness of biofilm formed by *C. albicans* (Figure 8 B), suggesting a direct role for shed Msb2 in adhesion and thus highlighting an important link between *C. albicans* virulence and Msb2 processing. The fact that shed Msb2 is heavily glycosylated (Figure 1 B and [18]) further bolsters this possibility. A role for Msb2 in *C. albicans* colonization further extends to limiting cell surface β-glucan exposure by Cek1 pathway as we observed greater β-glucan exposure in *msb2Δ/Δ* and *sap8Δ/Δ* strains (Figure 11 A). Also these phenotypes encompassing reduced biofilm formation and higher β-glucan exposure were comparable between *msb2Δ/Δ* and *sap8Δ/Δ* strains, suggesting a common defect responsible for these phenotypes (Figures 9, 10, & 11). Clinical reports also point to a central role of Saps in establishing *C. albicans* infection *in vivo*. Biofilms collected from clinical isolates of denture stomatitis patients had specifically higher expression of *SAP8* [37]. Furthermore, PA inhibited *C. albicans* invasion into epithelial and intestinal cells [38] and there is evidence that inhibitors of HIV proteinase that are administered as a part of the highly active anti-retroviral treatment (HAART) for HIV-positive patients, directly affect Saps' activity and reduce the incidence of oral candidiasis, independent of CD4^+^ T cell count [39]. Based on these observations, we propose that a major role of Sap proteins in facilitating virulence in *C. albicans* may largely be dependent upon their ability to process Msb2 that allow for Cek1 MAPK activation.

**Figure 12:**
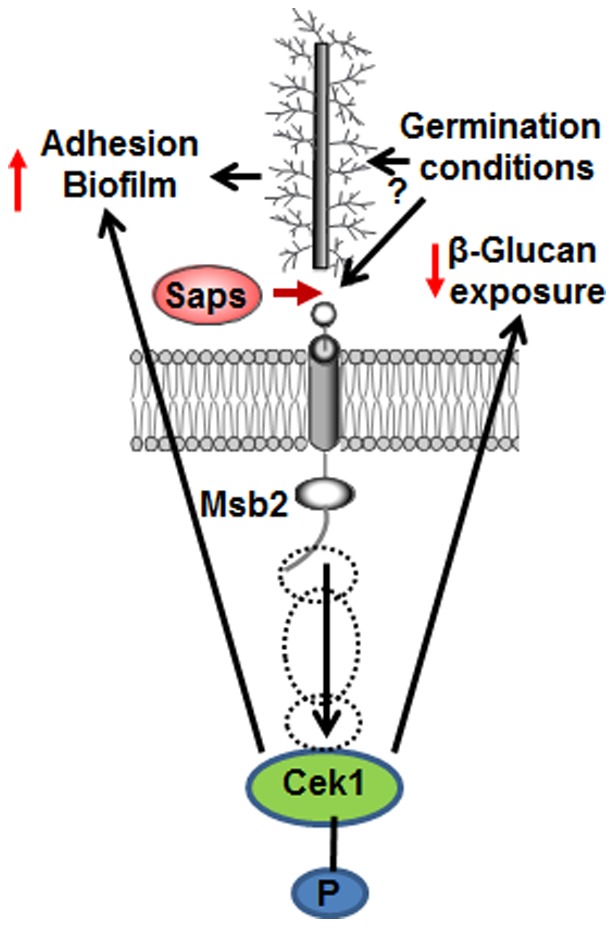
Model for Msb2 cleavage-dependent control of Cek1 mediated virulence traits in *C. albicans*. In the presence of appropriate signals (particularly germination conditions), secreted aspartic proteases-mediated cleavage of Msb2 is induced. This leads to release of the inhibitory, heavily glycosylated extracellular domain (shown here as an elongated bar with filaments) of Msb2, that in turn allows for Cek1 phosphorylation. As a result, virulence traits maintained by the Cek1 pathway, such as exposure of cell surface immunogens (β-glucan) and enhanced cell adhesion and biofilm formation abilities, are expressed at levels necessary for robust virulence. Dotted circles represent other members of this signaling cascade that are necessary for the transmission of signals from Msb2 to Cek1.

We present a model (Figure 12) linking proteoytic processing of signaling mucin Msb2 by Saps to virulence in *C. albicans*. We show that Msb2 cleavage occurs in response to specific environmental cues and is necessary for the activation of the Cek1 MAPK pathway. This cleavage controls germination, biofilm formation, and surface β-glucan exposure, three important virulence factors for *C. albicans*. It is not clear whether these cues affect Msb2 protein structure or modulate the activity of Sap enzymes, or both, in order to make the cleavage process amenable to the changes in the cell exterior. Thus, both Msb2 and Msb2-processing Saps may present promising opportunities as potential drug targets against candidiasis.

## Materials and Methods

### Strains and media

CAI4 (*Δura3::imm434/Δura3)* was used as the WT strain in this study. The knock-out mutant of Msb2 was constructed in CAI4 parental background using the *URA* blaster strategy as detailed previously [40], generating *Δura3::imm434/Δura3::imm434, Δmsb2::FRT*. Ura was placed at the *RPS10* locus to generate URA^+^ constructs (*msb2Δ/Δ*). For restoration strain construction of Msb2 (*msb2Δ/Δ+*), a copy of WT *MSB2* was introduced into the *RPS10* locus [41] of the *msb2Δ/Δ* strain (*URA* ^–^ version), using a previously described strategy [40]. For tagging the *MSB2* gene with an HA epitope, a PCR primer pair was designed to amplify a 3HA-URA3–3HA encoding region from the plasmid pCaMPY-3×HA [42]. These primers consist of 5` tail corresponding to sequence homologous to the flanking region of *MSB2* gene, from 776 to 882 bp which was substituted for four tandem repeats region within Msb2. The purified PCR product was transformed using Frozen-EZ Yeast Transformation II Kit™ (Zymo Research, CA, USA) into *MSB2* heterozygous strain (single allele knockout of *MSB2*). Transformed cells were spread onto uracil-deficient agar plates for selection of URA^+^ colonies, generating a URA^+^ MSB2-HA strain. *C. albicans* genomic DNA was isolated, and integration of the HA-CaURA3-HA cassette at the target position was confirmed by PCR, and the presence of the HA tag was confirmed by immunoblotting with HA-probe (F-7) antibody (Santa Cruz Biotech). All *sap* knockouts were constructed using the URA-blaster protocol, as described previously [43]–[46]. *sap8Δ/Δ* was constructed similarly to generate *SAP8*::*hisG*::*URA3*::*hisG::SAP8* strain. *SAP8* restoration strain (*sap8Δ/Δ+)* was made by recycling the *URA* marker from *sap8Δ/Δ*, followed by the insertion of *SAP8* along with *URA* at the *RPS10* locus. All the strains and their respective phenotypes are summarized in Table 1.

YPD was used to grow overnight cultures at 30°C to an OD_600_  = 2. For germination studies, overnights were diluted to an OD_600_ of 0.3 in prewarmed YNB media with 1.25% *N*-Acetyl-D-Glucosamine (NAG) as carbon source and grown at 37°C for indicated time periods, unless otherwise stated. For non-germination conditions, YNB media with 1.25% Glucose as carbon source was used with incubation at 30°C for desired time intervals. Candicidal assays were performed as described previously [47].

### Microscopic studies

#### Msb2 cell surface localization

Cells grown overnight in YPD medium were diluted to OD_600_  = 0.3, then cultured to an OD_600_  = 1 at 30°C. After this, cells were incubated at 37°C for 1 h. Samples were removed at indicated time intervals and incubated with anti-HA Alexa Flour 488 conjugate (Life Technologies), washed twice in PBS, and visualized at 40X magnification using an Axio Fluorescence Microscope.

#### Calcoflour white (CFW) staining

Cells grown overnight in YPD medium were diluted to OD_600_  = 0.3, then allowed to reach an OD_600_  = 1 at 30°C. After this, cells were incubated at 30°C for yeast cell analysis or 37°C for hyphae analysis for 1 h. Cells (10^6^) were then stained with 5 µl of Calcofluor white (Sigma-Aldrich) containing Calcofluor white (1 g/l) and Evans blue (0.5 g/l) and visualized microscopically using an Axio Fluorescence Microscope

#### Germination

Germination percentage was calculated among 200 cells grown under germination conditions and observed at 10X magnification under a light microscope.

### Protein and RNA work

#### Determination of levels of activated MAPK and total Msb2

Cells were grown, lysed, and blotted using antibodies to HA or phosphorylated Cek1p. Briefly, cell pellets were placed on ice and resuspended in 300 μl 10% TCA buffer (10 mM Tris HCl pH 8.0, 10% trichloroacetic acid, 25 mM NH4OAc, 1 mM Sodium EDTA). Total cellular lysate were isolated by disrupting cells with acid washed beads by vortexing for 1 min x 10 cycles using FastPrep®-24 Instrument (MP Biomedicals LLC). Samples were placed on ice for 5 min between each cycle. The acid washed beads were removed and the samples were centrifuged at 4°C for 10 min at 13000 rpm. The supernatant was removed and 150 μl of resuspension buffer (0.1 M Tris HCl pH 11.0, 3% SDS) was added. Samples were boiled for 5 min then centrifuged at 13000 rpm for 30 sec. Normalized protein content (20 µg) was separated by SDS-PAGE on 12% gels and transferred to nitrocellulose membranes. For Cek1p and Mkc1 phosphorylation, α-phospho p42/44 MAPK ERK1/2 Thr202/Tyr204 rabbit monoclonal (Signaling Technology) antibody was used as the primary antibody. Goat α-rabbit IgG-HRP (Jackson ImmunoResearch Laboratories, Inc.) was used as the secondary antibody. After transfer, membranes were incubated with primary antibodies at 4°C for 16 h in 5% BSA buffer (0.5 g BSA, 10 ml TBST), followed by washing with TBST. The membranes were then incubated with secondary antibodies at 25°C for one hour in blocking buffer, washed, and used for detection using SuperSignal West Pico detection kit (Thermo Scientific). Anti-HA antibody was used for the detection of HA-tagged Msb2, followed by detection using ECL plus chemiluminescence kit (Thermo Scientific).

#### Determination of Msb2 levels in cell wall and cytoplasmic fractions

The cell wall and cytoplasmic fractions were prepared as described by Ebanks *et al*. [48], with some modifications. After harvesting, cells were washed twice with chilled NaPB (pH 7.4) and resuspended in chilled lysis buffer (10 mM Tris, pH 7.4, 1X Protease inhibitor cocktail, Sigma-Aldrich). The cells were disrupted as detailed above. Cell disruption was confirmed microscopically for more than 90% cell breakage. Lysed cells were separated from the glass beads and centrifuged at 13000 rpm for 10 min. The cytoplasmic fraction was removed and kept at −80°C till further use. The cell wall pellet was further washed sequentially with chilled water, 5% NaCl, 2% NaCl, 1% NaCl, and finally with water again to remove any cytoplasmic fraction contamination. Cell wall proteins were extracted by boiling with extraction buffer (50 mM Tris, pH 8.0, 2% SDS, 5% β-ME, 0.1 M EDTA) for 10 min, centrifuged at 13000 rpm, and supernatant was kept at −80°C till further use. Msb2 was detected in these fractions as described.

#### Msb2 shedding in liquid cultures

For determination of Msb2 shedding under environmental stress conditions in liquid culture, overnight grown cells were diluted to an OD_600_  = 0.3 and allowed to grow to an OD_600_  = 1.0 under germination and non-germination conditions. The cells were harvested, washed once with sterile PBS, and resuspended in fresh media with or without stress agents (Sorbitol [1 M], NaCl [1 M], CFW [100 µg/ml], and H_2_O_2_ [5 mM]) for 1 h. Supernatants were collected and slot blotted with anti-HA antibody.

#### Msb2 shedding on solid surfaces and from biofilms

Overnight grown Msb2-HA strain was diluted to OD_600_  = 1.0, spotted directly on sterile nitrocellulose membrane which had been place on YNB+1.25%NAG+1% agar media and incubated at 37°C. Colonies were allowed to grow for 72 h and documented. The membrane was washed with TBS +0.05% tween 20 to remove the colonies and probed by immunoblotting using HA-probe. Different stress agents were added to the agar media in order to determine their effect on shedding on Msb2 at solid surface. The intensity of shed Msb2 was determined using Image J software. The relative Intensity was calculated by dividing intensity of shed Msb2 under different stress conditions with shed Msb2 under YNB.

The shedding of Msb2 in biofilm was determined by slot blot of the supernatant (normalized by biofilm mass) on the nitrocellulose membrane.

#### Reverse transcription PCR detection of MSB2 transcript levels

The relative levels of *MSB2* and control *18S* RNA were determined under given conditions as described previously [47].

### Biofilm studies

Biofilm formation for different strains was carried out using YNB +2% Glucose in 12-well polystyrene plates (BD Falcon). Strains were grown overnight in YPD at 28^°^C, washed, and diluted to OD_600_  = 1.0 in PBS. Cells (2 ml) were added to each well and the plates were incubated at 37°C for 2 h. Non adherent cells were removed by gentle washing of the wells and 2 ml media was added to each well. The plates were then incubated at 37°C for 24 h and 48 h to allow biofilm formation. For biomass measurements, medium was removed from the wells and 2 ml of PBS was added to each well to dislodge the biofilm by scraping. The content of each well was transferred to pre-weighed microfuge tubes, dried using a Speed-Vac, and weighed.

For Confocal microscopy, biofilm were allowed to grow on Lab-Tek chambered coverglass for 24 h as described above. Confocal images were acquired with a Zeiss LSM510 Meta Confocal Microscope (Carl Zeiss, Germany) using Plan Apochromat 63X/1.4 objectives. The depth was measured across the width of device at regular intervals. A series of horizontal (*x–y*) optical sections with a thickness of 0.38 μm were taken throughout the biofilm. The *Z*-stack images and thickness measurements were determined using Axio Vision 4.4 software (Carl Zeiss Micro Imaging).

### Assessment of surface β-glucan

Cells were grown under germination and non-germination conditions. Cells (3X10^6^) were incubated with anti-β-(1,3)-glucan monoclonal antibody (Biosupplies) at room temperature for 30 min, followed by incubation on ice for 20 min. Next, cells were washed twice with cold PBS. Cells were then incubated with Alexa-Fluor 647 conjugated secondary antibody (Cell Signaling Technology) for 30 min on ice and washed twice with the cold PBS. Cells were resuspended in 500 µl PBS and flow cytometry analysis was performed with FACSCalibur flow cytometry using CellquestPro Software (BD-Biosciences). Data analysis was performed using FCS Express 4 Flow Cytometry software (De Novo Software).

### Murine model of oropharyngeal candidiasis

We infected mice, using only URA^+^ *C. albicans* strains, in a murine model of oropharyngeal candidiasis as previously described [49]. The tongue and adjacent hypoglossal tissues of mice were excised after 5 days of infection and cut in half. One half was weighed and homogenized for quantification by CFU determination. The other half was processed for histopathological analysis by fixing in zinc-buffered formalin followed by 70% ethanol and then embedded in paraffin. Thin sections were cut and stained with periodic acid-Schiff.

### Ethics statement

This study was carried out in strict accordance with the recommendations in the Guide for the Care and Use of Laboratory Animals of the National Institutes of Health. The protocol was approved by the Institutional Animal Care and Use Committee of the University of Buffalo (Project Number: ORB06042Y). All infections were performed under anesthesia using ketamine and xylazine, and all efforts were made to minimize suffering.
