# Correction: Secreted Aspartic Protease Cleavage of *Candida albicans* Msb2 Activates Cek1 MAPK Signaling Affecting Biofilm Formation and Oropharyngeal Candidiasis

**DOI:** 10.1371/annotation/13ef5e14-c192-4bb2-af91-886024500b4b

**Published:** 2013-08-21

**Authors:** Sumant Puri, Rohitashw Kumar, Sonia Chadha, Swetha Tati, Heather R. Conti, Bernhard Hube, Paul J. Cullen, Mira Edgerton

A funding organization and grant were incorrectly omitted from the Funding Statement. The following sentence should be read after the first sentence of the Funding Statement: "P.J.C. is funded by the US Public Health Service grant GM098629." 

